# Evidence of traditional Chinese medicine for treating type 2 diabetes mellitus: from molecular mechanisms to clinical efficacy

**DOI:** 10.1080/13880209.2024.2374794

**Published:** 2024-07-19

**Authors:** Yadong Ni, Xianglong Wu, Wenhui Yao, Yuna Zhang, Jie Chen, Xuansheng Ding

**Affiliations:** a School of Basic Medicine and Clinical Pharmacy, China Pharmaceutical University, Nanjing, China; b Precision Medicine Laboratory, School of Basic Medicine and Clinical Pharmacy, China Pharmaceutical University, Nanjing, China

**Keywords:** Hypoglycemia, diabetes management

## Abstract

**Context:**

The global prevalence of type 2 diabetes mellitus (T2DM) has increased significantly in recent decades. Despite numerous studies and systematic reviews, there is a gap in comprehensive and up-to-date evaluations in this rapidly evolving field.

**Objective:**

This review provides a comprehensive and current overview of the efficacy of Traditional Chinese Medicine (TCM) in treating T2DM.

**Methods:**

A systematic review was conducted using PubMed, Web of Science, Wanfang Data, CNKI, and Medline databases, with a search timeframe extending up to November 2023. The search strategy involved a combination of subject terms and free words in English, including ‘Diabetes,’ ‘Traditional Chinese Medicine,’ ‘TCM,’ ‘Hypoglycemic Effect,’ ‘Clinical Trial,’ and ‘Randomized Controlled Trial.’ The studies were rigorously screened by two investigators, with a third investigator reviewing and approving the final selection based on inclusion and exclusion criteria.

**Results:**

A total of 108 relevant papers were systematically reviewed. The findings suggest that TCMs not only demonstrate clinical efficacy comparable to existing Western medications in managing hypoglycemia but also offer fewer adverse effects and a multitarget therapeutic approach. Five main biological mechanisms through which TCM treats diabetes were identified: improving glucose transport and utilization, improving glycogen metabolism, promoting GLP-1 release, protecting pancreatic islets from damage, and improving intestinal flora.

**Conclusions:**

TCM has demonstrated significant protective effects against diabetes and presents a viable option for the prevention and treatment of T2DM. These findings support the further exploration and integration of TCM into broader diabetes management strategies.

## Introduction

Diabetes mellitus is a metabolic disorder characterized by elevated blood glucose levels and clinical symptoms, including polydipsia, polyuria, emaciation, and fatigue. Type 2 diabetes mellitus (T2DM), comprising approximately 95% of these cases, is a major global health concern. In 2021, around 537 million adults aged 20 to 79 years lived with diabetes. Projections suggest that this number will rise to 643 million by 2030 and 783 million by 2045 (Gregory et al. 2022). The global crude prevalence rate of diabetes in the 20–79 age group is currently 10.5% and is expected to increase to 11.3% by 2030 and 12.2% by 2045 (Gregory et al. 2022). The age-standardized prevalence rate is projected to increase from 9.8% in 2021 to 11.2% by 2045 (Gregory et al. 2022). As an incurable chronic disease, uncontrolled diabetes mellitus leads to various complications, resulting in severe consequences, extensive medication use, high outpatient and inpatient expenses, and repeated diagnostic tests. T2DM, also known as non-insulin-dependent diabetes mellitus, not only induces hyperglycemia but also triggers multiple life-threatening complications such as diabetic retinopathy, diabetic nephropathy, and hyperlipidemia. It is the most common form of diabetes (Constantino et al. 2013; Bello et al. 2014). Given the increasing prevalence and proportion of diabetes in the population, the urgent need for effective therapeutic strategies to manage this condition cannot be overstated.

The management of hyperglycemia in diabetes is multifaceted, encompassing lifestyle management, glucose monitoring, diabetes education, and the use of hypoglycemic drugs. In terms of preventing T2DM, medications such as metformin, α-glucosidase inhibitors, orlistat, thiazolidinediones (glitazones), glucagon, and glucagon-like peptide-1 (GLP1) receptor agonists have shown efficacy (Haw et al. 2017). Oral hypoglycemic agents are categorized based on their mechanism of action: those that primarily stimulate insulin secretion, including sulfonylureas, glinides, and dipeptidyl peptidase-IV inhibitors (DPP-4i), and those that work through other mechanisms, including biguanides, thiazolidinediones (TZDs), α-glycosidase inhibitors, and sodium-glucose cotransporter-2 inhibitors (SGLT2i). Although pharmacological treatments are effective and cost-effective, prolonged use can lead to side effects and complications such as hypoglycemia, fluid retention, and edema (Diamant and Heine 2003). Therefore, the development of new, more moderate, cost-effective, and efficient therapeutic strategies beyond traditional hypoglycemic agents is imperative.

Traditional Chinese medicine (TCM), often regarded the ‘bright pearl’ within the realm of global medicine, has been a pioneer in the prevention and treatment of diabetes, demonstrating notable efficacy (Liu et al. 2004; Tong et al. 2012; Lu et al. 2020). In response to the increasing prevalence of diabetes both in China and globally, numerously clinical trials have been conducted, leading to the development of innovative herbal products (Lian et al. 2014, 2015; Pang et al. 2017). The use of these products in the treatment of chronic diseases such as diabetes not only broadens therapeutic options but also potentially improves patient quality of life. However, a thorough understanding of the properties of these plants and their scientific backing is essential. Previous reviews have examined the use of herbal medicines in T2DM, focusing on the therapeutic efficacy and biochemical mechanisms of various natural products (Huang et al. 2020; Sun et al. 2021b; Su et al. 2022). However, these studies often report preliminary findings or lack complete documentation, highlighting the need for further research and development. This review specifically focuses on the hypoglycemic effects of TCM, delving into recent advances in the application of TCM to manage hyperglycemia in T2DM.

## Methods

### Study selection

Based on evidence from randomized controlled trials (RCTs) and systematic reviews, we conducted searches in Web of Science, PubMed, Wanfang Data, China Knowledge Resource Integrated Database (CNKI), and Medline. The databases were searched up to November 2023 using a combination of subject terms and free words. English search terms included ‘Diabetes,’ ‘Traditional Chinese Medicine,’ ‘TCM,’ ‘Hypoglycemic Effect,’ ‘Clinical Trial,’ and ‘Randomized Controlled Trial.’

The inclusion criteria were as follows: (1) clinical and randomized controlled studies of traditional Chinese patent medicines to improve type 2 diabetes; (2) clinical and randomized controlled studies of empirical formulas to improve type 2 diabetes; (3) clinical and randomized controlled studies of Chinese medicine monomers to improve type 2 diabetes; (4) clinical trials and animal experiments that complied with ethical research requirements; (5) studies involving patients with type 2 diabetes or type 2 diabetes model animals; and (6) clinical trials using a multicenter, randomized, controlled, double-blind trial design.

Exclusion criteria were: (1) studies on the treatment of diabetic complications without hypoglycemic effects using traditional Chinese medicine; (2) clinical and randomized controlled studies of TCM combined with surgical treatments to improve type 2 diabetes; (3) randomized controlled trials of Chinese medicine combined with Western medicine or surgery without an individual treatment control group; (4) clinical trials and animal experiments that did not meet ethical research requirements; and (5) research focused on type 1 diabetes or gestational diabetes.

The literature screening was performed by two investigators who independently removed duplicates and initially reviewed the titles and abstracts to exclude studies that did not meet the criteria. They then read the full texts of the remaining articles to confirm their suitability. In cases of disagreement, a third investigator was consulted to decide after discussion. A total of 108 relevant articles were screened.

### Clinical efficacies and preclinical evidence of traditional Chinese medicines in diabetes treatment

#### Traditional Chinese patent medicines for diabetes treatment

Traditional Chinese patent medicines, which are standardized formulations approved by the National Medical Products Administration (NMPA) and commercialized by pharmaceutical companies, play an important role in the treatment of hyperglycemia. These drugs have fixed ingredients and dosages and are often used alone or in combination with other drugs to manage T2DM. Examples of such medicines include the compound Jinlida granule, Tianqi capsule, Tang-Min-Ling-Wan (TM81), Xiaoke pill, YH1 (comprising *Rhizoma coptidis* and Shen-Ling-Bai-Zhu-San), and JinqiJiangtang (JQJT). These examples are supported by studies referenced in the literature (Ji et al. 2013; Tong et al. 2013; Lian et al. 2014, 2015; Wang et al. 2017; Huang et al. 2019; Pan et al. 2021). Details are shown in Table 1.

**Table 1.** Chinese traditional patent medicines for the treatment of diabetes.

Name	Recipe composition	Manufacturer	Dosage	Duration (Week)	DOI
Jinlida granule	Danshensu sodium salt, Puerarin, salvianolic acid B, Epimedin B, Epimedin C, Icariin, and Ginsenosides Rb1, Rc, and Rb2	YiLing Pharmaceutical Co., Ltd., Hebei, China Pharmaceutical	9g per time, three times per day	12W	10.1155/2021/6303063
Tianqi capsule	Astragali Radix, Coptidis Rhizoma, Trichosanthis Radix, Ligustri Lucidi Fructus, Dendrobii Caulis, Ginseng Radix, Lycii Cortex, Ecliptae Herba, Galla Chinensis, and Corni Fructus	Baoquan Pharmaceutical Co., Ltd., Heilongjiang, China Pharmaceutical	Five capsules before each meal, three times per day	52W	10.1210/jc.2013-3276
Tang-Min-Ling-Wan	Rhizoma Coptidis, Radix Paeoniae Alba, Radix Scutellariae, Pericarpium Cirtri Reticulatae, Rhizoma Rhei	The Research Department	6g per time, 3 times per day	12W	10.1371/journal.pone.0056703
Xiaoke Pill	Rehmanniae, Radix Astragali, Radix Trichosanthis, Stylus Zeae Maydis, Fructus Schisandrae Sphenantherae, and Rhizoma Dioscoreae	Guangzhou Pharmaceutical Co., Ltd., Guangzhou, China	Five pills per day	48W	10.1111/dom.12051
YH1	Rhizoma copies (RC) and Shen-Ling-Bai-Zhu-San (SLBZS)	Sun Ten Pharmaceutical Co., Ltd. Taiwan, China	6g before each meal, three times per day	12W	10.1371/journal.pone.0221199
JinqiJiangtang tablets	Berberine, astragalus, and honeysuckle	Institute of Materia Medica of the Chinese Academy of Medical Sciences	Seven tablets before each meal, two times per day	84W	10.1038/s41598-017-11583-5

A prominent example of an effective traditional Chinese patent medicine is the Jinlida granule, a compound preparation widely used in clinical treatment of T2DM. In an RCT involving 192 T2DM patients, the combination of Jinlida granule and metformin significantly improved fasting plasma glucose (FPG) [mean deviation (MD) ± standard deviation (SD): (–1.34 ± 1.70) vs. (–0.73 ± 1.70)], 2 h postprandial glucose (2hPG) [MD ± SD: (–2.95 ± 3.99) vs. (–1.57 ± 4.34)], and HbA1c [MD ± SD: (–0.92 ± 1.09) vs. (–0.53 ± 0.94)] compared to metformin alone after a 12-week treatment period (Lian et al. 2015). These patients reported no serious adverse events, no adverse effects on the electrocardiogram (ECG), or abnormalities in routine blood, liver, or renal functions at the 12-week mark. Similarly, another RCT with 158 T2DM patients demonstrated significant improvements in FPG, 2hPG, and HbA1c with the same combination over 16 weeks (*p* < 0.01) (Pan et al. 2021). These trials have established the Jinlida granule as a safer and more effective option than metformin monotherapy, particularly when used as an adjunctive treatment for T2DM.

Recent clinical studies have also highlighted the preventive effects of traditional Chinese patent medicines on T2DM. The Tianqi capsule, a new Chinese medicinal formulation consisting of 10 different herbs, has shown promise in the treatment of T2DM. In a recent RCT involving 420 patients with 2hPG levels between 7.8 and 11.1 mmol/L after an oral glucose tolerance test (OGTT) and fasting blood glucose greater than 7.0 mmol/L, Tianqi capsule reduced the risk of diabetes by 32.1% compared to a placebo (Lian et al. 2014). This significant finding underscores its considerable preventive impact on the progression of T2DM.

JinqiJiangtang (JQJT tablets), the first Chinese patent medicine approved by the NMPA specifically for diabetes (Vray and Attali 1995), has demonstrated preventive capabilities against T2DM. The components of JQJT may enhance insulin resistance by modulating glucolipid metabolism, reducing oxidative stress, and improving immune function. In an RCT involving 362 pre-diabetes patients, the progression to diabetes was significantly lower in the JQJT group compared to the placebo group (16.5% vs. 28.9% at 12 months, *p* = 0.005; 20.3% vs. 32.8% at 24 months, *p* = 0.007) (Wang et al. 2017).

The Xiaoke pill has been shown to effectively reduce the risk of hypoglycemia and improve glycemic control in T2DM patients with inadequate glycemic management. This was demonstrated in a multicenter, non-inferiority, double-blind, controlled trial that compared its effects with glibenclamide over 48 weeks. Similarly, YH1 and TM81 have demonstrated efficacy in managing diabetes in separate RCTs (Tong et al. 2013; Huang et al. 2019).

These RCTs suggest that traditional Chinese patent medicines are potent in improving diabetes-related parameters such as FPG, 2hPG, and HbA1c, while also reducing the prevalence of T2DM. Their safety profile is generally acceptable in many clinical practices.

#### Empirical formulas for diabetes treatment

In TCM, practitioners often devise empirical formulas grounded in the theories of Chinese medicine. These formulas are typically derived from ancient medical texts or from clinical experiences of experienced physicians. They are customized by adding or subtracting herbs and adjusting the dosages to suit the unique physical condition of each patient. Although some of these formulas achieve a degree of standardization in terms of composition, dosage, and preparation, they are not commercially available and are used exclusively in the hospitals where they are produced. Details of these empirical formulas are provided in Table 2.

**Table 2.** Chinese empirical formulas for the treatment of diabetes.

Name	Recipe composition	Dosage	Duration	DOI
Tonifying-qi-nourishing-yin-activating-blood decoction	Radix Panax Ginseng, Rhizoma Atractylodis Macrocephalae, Radix Rehmanniae, Radix Puerariae, Radix Astragali, Salviae Miltiorrhizae, Rhizoma Dioscoreae, Rhizoma Smallpox Powder, Radix Paeoniae Lactiflorae, Radix Angelicae Sinensis, Rhizoma Ligustici Chuanxiong.	One dose is divided into two equal portions per day, two times per day.	1M	10.16658/j.cnki.1672-4062.2021.20.029
Decoction for replenishing Qi, nourishing Yin, activating Blood, and removing Blood Stasis	Rhizoma Ligustici Chuanxiong, Radix Panax Ginseng, Radix Panax Ginseng, Rhizoma Maitake, Salviae Miltiorrhizae 10 g each, Radix Rehmanniae Praeparata, Cornu Cervi Pantotrichum 15 g, Rhizoma Dioscoreae, Astragalus Membranaceus 30 g each, Rhizoma Dioscoreae, Rhizoma Dioscoreae, Cangzhu, Pueraria Mirifica	One dose is divided into three equal portions per day, three times per day	NA	10.15912/j.cnki.gocm.2017.08.172
Jia Jian Di Dang Decoction	Fahanxia, Rhizoma Atractylodis Macrocephalae, leech, peach kernel, ripe rhubarb, Poria Cocos, Pericarpium Citri Reticulate	One dose per day after lunch	8W	10.15912/j.cnki.gocm.2015.23.150
Baihu Renshen decoction	Radix Panax Ginseng (20g), Gypsum Cauliflower (20g), Rhizoma Polygonati Odorati (8g), Rhizoma Dioscoreae (20g), Rhizoma Smallpox Powder (5g), Radix et Rhizoma Glycyrrhizae Praeparata(3g)	One dose per time, two times per day	4W	https://kns.cnki.net/kcms2/article/abstract?v=Jk1LZv7y6P2yXGaAC6HDu_EsrQIhf6zsYhNw979-ps6rZzlezvR5c1lVKt6kShSc6p153app3ZHUE-Hh-wgs7VOUSvO0ZvGo3M4qTT-ts_RoBFZ_45qYluFkygzwQoFmCe9Gfwi_r_I=&uniplatform=NZKPT&language=CHS
Jianpi Huashi Decoction	Radix Panax Ginseng, Fenghuang Cao 30 g each, Poria 25 g, Radix Rehmanniae Praeparata, Poria 20 g each, Radix et Rhizoma Glycyrrhizae Praeparata, Pericarpium Citri Reticulate, Herba Epimedium 10 g each, Citrus Aurantium Lepidum 6 g	One dose per time, two times per day, before meals	3M	https://kns.cnki.net/kcms2/article/abstract?v=Jk1LZv7y6P0f7oNsSLLVDBl1tvwCTbbZy80_5BFVQga81XxfDU6nnWa_qKRPz_j78UvpchlCysDoqfoQyWwdl6-lyiAWzM-n5otJFAy56XR0WfeokzSLmHobL-w_0C56Qct0wVpndaA=&uniplatform=NZKPT&language=CHS
Gegen Qinlian Decoction (GQD)	Ge Gen (Radix Puerariae), Huang Qin (Radix Scutellariae), Huang Lian (Rhizoma Coptidis), Zhi Gan Cao (Radix Glycyrrhizae) and Gan Jiang (Rhizoma Zingiberis).	One dose per time, two times per day, before meals	12W	10.1016/s0254-6272(11)60013-7

Among the empirical formulas commonly used for diabetes treatment are Jia Jian Di Dang decoction, Tonifying-qi-nourishing-yin-activating-blood decoction, Baihu Renshen decoction (BRD), Gegen Qinlian decoction (GQD), and Jianpi Huashi decoction (JHD) (Tong et al. 2011; Duan et al. 2015; Xu, Lian, et al. 2015; Liu 2017; Yang 2017; Liu and Xu 2021; Qiu et al. 2023). The tonifying-qi-nourishing-yin-activating-blood decoction, developed through extensive clinical research and experience in TCM, has demonstrated significant clinical efficacy in both preventing and treating diabetes and its complications. In a clinical study involving 96 T2DM patients, this decoction, used in combination with pitavastatin, significantly improved FPG, 2hPG, and HbA1c levels compared to pitavastatin alone (*p* < 0.05) (Liu 2017). Another study with 90 T2DM patients reported similar improvements in FPG, 2hPG, HbA1c, fasting insulin (FINS), and homeostasis model assessment of insulin resistance (HOMA-IR) when the decoction was combined with metformin (*p* < 0.05) (Liu and Xu 2021).

Baihu Renshen decoction (BRD), based on the ‘Treatise on Febrile Diseases,’ has been the focus of recent studies exploring its therapeutic properties. These studies emphasize the role of quercetin and anemarrhena in BRD, noting their potential to restore pancreatic function and provide antioxidation and hypoglycemic effects. An RCT published by Qiu et al. (2023) demonstrated the efficacy of BRD in reducing FPG and 2hPG levels compared to baseline (*p* < 0.05). There was a significant difference in the decrease in total effective rate between the BRD treatment group and the control group, with 85% effectiveness in the BRD group compared to 65% in the control group (*p* < 0.05).

In TCM, combining empirical prescriptions with insulin therapy has shown promising results in clinical trials. An RCT conducted by Duan et al. (2015) assessed the efficacy of combining insulin with the Jia Jian Di Dang decoction. This combination was more effective than insulin monotherapy, particularly in improving FPG and HbA1c levels in patients with poorly controlled blood glucose levels (*p* < 0.05). Additionally, Jia Jian Di Dang decoction significantly improved insulin resistance, as evidenced by an increase in HOMA-IR from 2.13 ± 0.06 to 3.73 ± 1.10 (*p* < 0.05).

Another RCT by Yang (2017) compared the effects of Jianpi Huashi decoction (JHD) with insulin therapy in 86 T2DM patients. The study found that JHD reduced HbA1c, FPG, and 2hPG levels more effectively in T2DM patients compared to insulin therapy alone (*p* < 0.05). These studies suggest that combining insulin with empirical prescription medications yields greater efficacy than insulin monotherapy.

The Ge Gen Qin Lian decoction (GQD), a traditional Chinese herbal formula dating back to the eastern Han dynasty and primarily used by the renowned physician Zhang Zhongjing (150–219 AD) to treat diarrhea, has also shown potential in diabetes treatment. An RCT by Tong et al. (2011) involving 54 T2DM patients treated with GQD observed a reduction in HbA1c of 0.66% from an initial level of 8.33% with a medium dose (clinical equivalent dose). Another randomized, double-blind clinical trial by Xu, Lian, et al. (2015) involving 187 T2DM patients showed that GQD significantly reduced FPG and HbA1c in a dose-related manner compared to placebo. GQD also induced dose-dependent changes in the gut microbiota, suggesting a correlation between these structural changes and its antidiabetic effects.

#### Chinese medicine monomer for diabetes treatment

With the ongoing advances in Chinese medicine research, a significant number of herbal extracts and individual components have been identified for their potential to improve diabetes management. This review focuses on studies with well-defined ingredients, dosages, and comprehensive efficacy records. Table 3 summarizes some of the most researched monomers and extracts in the treatment of T2DM.

**Table 3.** Chinese medicine monomer for the treatment of diabetes.

Active Compounds	Medicinal Materials	Dosage	Duration (week)	DOI
Sangzhi alkaloids(SZ-A)	Mulberry twigs	50mg per time, three times per day.	4W	10.2337/dc20-2109
*Lycium barbarum* polysaccharide (LBP)	Lycium barbarum	150mg per time, two times per day	12W	10.2174/1573406410666141110153858
*Trigonella foenum graecum* L. total saponins (TFGs)	Fahanxia, Rhizoma Atractylodis Macrocephalae, leech, peach kernel, ripe rhubarb, Poria Cocos, Pericarpium Citri Reticulate	Six capsules per time, three times per day	12W	10.1007/s11655-007-9005-3
Berberine	Coptis chinensis French	500mg per time, three times per day.	12W	10.1016/j.metabol.2008.01.013

Previous research (Mudra et al. 2007; Chan et al. 2016) has highlighted the hypoglycemic effects of certain herbs derived from parts of the mulberry tree. SZ-A, an herbal preparation composed primarily of alkaloids from mulberry branches, has demonstrated the ability to decrease both fasting and non-fasting glucose levels, enhance insulin resistance, and stimulate glucose-stimulated insulin secretion. In an RCT conducted by Qu et al. (2021) involving 517 T2DM patients, SZ-A was compared to acarbose, which served as a positive control. The trial, lasting 24 weeks, showed that both SZ-A and acarbose significantly reduced FPG, 1 h postprandial blood glucose (1hPBG), and 2 h postprandial blood glucose (2hPBG) from baseline levels (*p* < 0.001). SZ-A exhibited a hypoglycemic effect comparable to that of acarbose. Furthermore, safety assessments (Qu et al. 2021) did not reveal severe adverse reactions related to SZ-A, and the incidence of treatment-associated events (TAEs) and gastrointestinal disturbances was significantly lower with SZ-A than with acarbose (*p* < 0.001).

The *Lycium barbarum* polysaccharide (LBP), identified as an active component of *Lycium barbarum* L. (Solanaceae), has received significant attention in diabetes research. Several cellular and animal studies (Jin et al. 2013; Zhu et al. 2013) have shown that LBP significantly reduces blood glucose levels and improves insulin sensitivity. This is achieved by promoting glucose metabolism and insulin secretion, as well as stimulating the proliferation of pancreatic β-cells. In a randomized, double-blind, placebo-controlled trial involving 67 T2DM patients, LBP capsules exhibited greater protective effects compared to a placebo after a 3-month intervention. Improvements were observed in the area under the curve (AUC) for glucose and the insulinogenic index (*p* < 0.05). Additionally, LBP was found to reduce the AUCs for glucose, insulin, and HOMA-IR in patients who did not take hypoglycemic medication (*p* < 0.05). However, its efficacy was less pronounced in patients already taking hypoglycemic drugs (Cai et al. 2015).

*Trigonella foenum-graecum* L. (Fabaceae) total saponins (TFGs), extracted from fenugreek, have also been shown to possess hypoglycemic properties (Lu et al. 2008). A clinical study with 69 T2DM patients, whose blood glucose levels were inadequately controlled by oral hypoglycemic sulfonylurea drugs, demonstrated that a combination of TFGs and sulfonylureas significantly reduced FPG, 2hPBG, and HbA1c after a 12-week treatment period compared to sulfonylurea monotherapy (*p* < 0.05 or *p* < 0.01).

Berberine, the main active component of *Coptis chinensis* Franch (Ranunculales), an ancient Chinese herbal medicine, has been used for millennia to treat diabetes. An RCT conducted by Yin et al. (2008) involving 36 T2DM patients compared the effects of berberine with those of metformin. The study found that the efficacy of berberine in regulating glucose metabolism, including HbA1C, FPG, postprandial blood glucose (PBG), fasting insulin, and postprandial insulin, was comparable to that of metformin (*p* < 0.01). Berberine demonstrated superior effectiveness in regulating lipid metabolism compared to metformin. In another RCT by the same authors that included 48 T2DM patients, the additive or synergistic effects of berberine were assessed when combined with classic anti-diabetic agents. This 5-week combination therapy significantly reduced HbA1c levels from 8.1% to 7.3% (*p* < 0.01).

A clinical study by Gu et al. (2010) involving 60 T2DM patients used comprehensive metabolomics methods to analyze serum samples before and after treatment. The results indicated that berberine administration led to a significant reduction in the concentrations of 13 fatty acids, suggesting that berberine may play a crucial role in T2DM treatment by down-regulating high levels of free fatty acids.

#### Preclinical evidence for the treatment of diabetes with TCM

Numerous herbs have demonstrated direct or indirect hypoglycemic effects in preclinical studies, showing potential for development as effective treatments for hyperglycemia. Examples include *Arctium lappa* L. (Asteraceae), *Penthorum chinense* Pursh. (Penthoraceae), Shenglian decoction (SL), ginsenoside Rb1, tea polysaccharide, *Morus alba* L. (Moraceae) fruit polysaccharides, and Korean pine nut protein (PNP), as detailed in Table 4.

**Table 4.** Preclinical evidence for the treatment of diabetes with TCM.

Intervention	Subjects	Dosage and duration	Outcome	DOI
Ginsenoside Rb1	KK-Ay Mice	200 mg/kg/day; Two weeks	The RBG levels (*p* < 0.05) and FBG levels (*p* < 0.05) were obviously decreased. AUC values of OGTT test (p < 0.05) were reversed by administration of Rb1. The ginsenoside Rb1 group administration reversed the serum insulin (*p* < 0.05) and HOMA-IR (*p* < 0.05) compared with Kkay group. Islet morphology was more complete, and islet cell hypertrophy was relieved, which was consistent with metformin-treated mice.	10.1016/j.jep.2022.115997
*Lycium barbarum* L.	Rats	1.04g/kg/d and 2.08g/kg/d, respectively;4 weeks	The FBG levels were reduced by 19.5% and 19.9%, respectively, after administration of high and low doses of LLB extract compared to the control group. The HbA1c levels (*p* < 0.05) and FBG levels (*p* < 0.05) were obviously decreased.	10.1016/j.biopha.2019.109559
Shorea roxburghii Leaf Extract	SD Rats	100mg/kg/d and 400mg/kg/d, respectively;4 weeks	The weight gain in the SRL400 treated group (*p* < 0.05) after the intervention period. The food and fluid intake were also observed to be significantly reduced in the rats treated with SRL compared to the diabetic control (DCG) groups (*p* < 0.05).	10.1002/cbdv.201900661
Gegenqinlian decoction (GQD)	SD Rats	1.35g/Kg/day;4 weeks	The weight of rats in the Sax, GQD, and Sax + GQD groups increased significantly (*p* < 0.05, *p* < 0.01) after four weeks of administration. The FBG level between the treatment and MC groups differed significantly (*p* < 0.05).	10.1002/bdd.2374
Arctigenic acid (AA)	GK Rats and Wistar Rats	50mg/kg; two times per day.12 weeks	At the end of the experiment, AA decreased the FBG levels by 37.6% while nateglinide decreased that by 28.1% as compared to the model group; The HbA1c value of rats in both nateglinide (*p* < 0.01) and AA (*p* < 0.01) groups were lower than rats in the model group significantly. Compared with the model group, the plasma 2Hpg levels of rats treated with nateglinide and AA were reduced by 22.0% and 33.7% (*p* < 0.01), respectively, compared to their diabetic control counterparts. The treatments of nateglinide and AA restored the irregularity of islets to the structure similarly to that of normal control samples.	10.1016/j.phymed.2014.11.006
Tea polysaccharides (Acidic Tea Polysaccharides from Yellow Leaves of Wuyi Rock Tea)	Wistar Rats	200mg/kg/d,400mg/kg/d and 800mg/kg/d, respectively;40 days	The blood glucose values of diabetic rats after 40 d of tea polysaccharide intervention (MT group) were reduced, and the difference was statistically significant when compared with the DC group (*p* < 0.05). The tea polysaccharide treatment groups had less β-cell destruction, and all of the tea polysaccharide treatments improved the distribution, cell morphology, and staining granule distribution within the islets to different degrees.	10.3390/foods11040617
Shenlian (SL) Decoction	C57BL/KsJ-db/db Mice	4.55 g /kg/day and 0.455 g /kg /day respectively;8 weeks	Metformin, HL, RS, and SL decoction significantly lowered blood glucose in the first four weeks (*p* < 0.01, 0.05, 0.01, and 0.05). HL could improve the insulin generation in islet tissue (*p* < 0.05)	10.1155/2022/7802107
Aqueous Extract of *Trichosanthes kirilowii* Maxim (TKE)	BALB/c Mice and C57BL/6J Mice	500mg/kg/d,1000mg/kg/d and 2000mg/kg/d, respectively;NA	The hypoglycemic activity of TKE displayed a dose-dependent manner, and the inhibition reached 26.4 ± 3.19% at 2g/kg. TKP was able to activate Insulin receptor (IR) kinase activities.	10.1186/s12906-017-1578-6
Korean pine nut protein (PNP)	Kunming Mice	125mg/kg/d,250mg/kg/d and 500mg/kg/d, respectively;4 weeks	Compared with the DM group, the average food intake level of the PNP-M group decreased by 22.3%. The increase of FBG in each treatment group compared with the DM group was significantly inhibited (*p* < 0.05). After 3 weeks, the PNP-M group had the most significant hypoglycemic effect (*p* < 0.05). The insulin sensitivity of each treatment group significantly improved over the DM group (*p* < 0.05).	10.1016/j.biopha.2019.108989
Acidic hetero chain polysaccharides (CTP3-B, CTP3-C, and CTP3-D)	KK-Ay Mice	40mg/kg/d, 80mg/kg/d and 160mg/kg/d, respectively;4 weeks	CTP3 treatments (LP, MP, and HP) significantly (*p* < 0.05) suppressed the body weight gain in a dose-dependent way from week two, like the effects of Acarbose (PC). High-dose CTP3 treatment (HP) effectively decreased the daily dietary intake of KK-Ay mice (*p* < 0.05). In the treatment groups, blood glucose levels were lower than the DC at 30, 60, and 120min, especially in the MP and HP groups at 120min (*p* < 0.01).	10.1080/14786419.2020.1756805
Isodon rubescens	SD Rats	1.32g/kg/day;4 weeks	Compared to rats in the model group, the FBGs of rats in the positive(metformin (0.18 g/kg) by gavage) and water extract groups decreased significantly (*p* < 0.05).	10.1055/a-1147-9196
*M. alba* L. fruit polysaccharides (MFP50 and MFP90)	Wistar Rats	400 mg/kg/day;7 weeks	The glycated serum protein (GSP) levels were reduced by 18.6% (*p* < 0.01), 31.7% (*p* < 0.01), and 29.0% (*p* < 0.05) in the MFP50, MFP90. Relative to the DM group, the Fasting serum insulin (FINS) of both the MFP50 (*p* < 0.05) and MFP90 (*p* < 0.01) groups were significantly reduced. The HOMA-IR values of the MFP90 group (4.35 ± 1.62) were markedly lower than that in the metformin group (4.70 ± 2.43). MFP50 and MFP90 showed better effects than the metformin group in terms of the recovery of STZ-induced impairment of pancreatic islets (40 mg/kg).	10.1016/j.jep.2017.02.003
*Penthorum chinense* extract	Wistar Rats	150mg/kg/d and 300mg/kg/d, respectively;2weeks	Treatment with PCI and PCII caused a significant reduction (*p* < 0.05, *p* < 0.01) in blood glucose levels by 24.87% and 24.10% in the 1st second week, respectively. PCII significantly (*p* < 0.05) prevented the increase in blood glucose levels at 60 min and 120 min. Insulin levels in diabetic rats were significantly increased by treatment with Penthorum chinense extract (300 mg/kg) (*p* < 0.05).	10.1016/j.jep.2015.01.014
Combined extracts of *Scutellaria baicalensis* Georgi (SR) and *Coptis chinensis* Franch. (CR)	SD Rats	6.3 g/kg/day; NA	The level of FBG in T2DM rats was significantly reduced by SR and CR, especially SC. The activity of FBPase in T2DM rats was remarkably inhibited after oral administration of SR, CR, and especially SC. The activities of GK, PFK, and PK were markedly increased by SC.	10.1002/jssc.201801204
Hawthorn (*Crataegus pinnatifida* Bge)	SD Rats	50mg/kg,100 mg/kg, and 200mg/kg, respectively; two times per day;2weeks	The level of FBG in T2DM rats was significantly reduced by Hawthorn extract at high, middle, and low doses (*p* < 0.01 and *p* < 0.05). Serum insulin levels were increase by 206.76 ± 3.76, 204.05 ± 15.17 and 176.82 ± 1.45 pg mL-1, compared to the T2DM group respectively. (*p* < 0.01).	10.1002/jsfa.8323

A recent study revealed a significant improvement in glucose tolerance in glucose fed hyperglycemic GK rats, showing that arctic acid reduced plasma glucose and fasting blood glucose levels by 37.6% and 28.1%, respectively, compared to the control group (nateglinide) (Xu, Gu, et al. 2015). The results indicate that arctic acid was more effective than nateglinide in reducing islet irregularities and lowering blood glucose levels.

*Penthorum chinense*, another herb with a mechanism similar to those previously discussed, exhibits notable anti-hyperglycemic activities. This efficacy is demonstrated by decreased serum HbA1c, triglycerides (TG), total cholesterol (TC), and low-density lipoprotein cholesterol (LDL-C), along with increases in high-density lipoprotein cholesterol (HDL-C) and insulin levels. A study revealed that treatment with *Penthorum chinense* extract (300 mg/kg) significantly reduced blood glucose levels by 29.42% and 22.61% in the first and second weeks, respectively, compared to the acarbose group (12 mg/kg), which experienced reductions of 8.17% and 10.45% in the corresponding weeks (*p* < 0.05). Additionally, this treatment significantly increased insulin levels in diabetic rats (*p* < 0.05). Diabetic rats treated with *Penthorum chinense* extract also showed significant increases in superoxide dismutase (SOD) and L-glutathione (GSH) activities, and a decrease in serum malondialdehyde (MDA) levels, compared to diabetic control rats (Huang et al. 2015). These findings suggest that the antidiabetic effect of *Penthorum chinense* extract may be related to its antioxidant properties, attributed to polyphenols.

Hawthorn (*Crataegus pinnatifida* Bge. [Rosaceae]), a traditional medicinal plant that is long used in folk medicine and pharmaceutical preparations, is recognized for its hypoglycemic effects, which may be attributed to the abundant polyphenolic compounds it contains (Aierken et al. 2017).

Several studies have underscored the role of TCM formulations in exerting hypoglycemic effects through modulation of intestinal flora. In an eight-week study, mice were allocated into five groups, each receiving different treatments: distilled water, *Coptis chinensis* (HL), *Panax ginseng* C.A.Mey. (Araliaceae) (RS), Shenlian (SL) (a combination of *Coptis chinensis* and *Panax ginseng*) and metformin, respectively (Sun et al. 2022). After the treatment period, HL, RS, and SL decoctions successfully reversed decreased microbiota diversity and richness. Specifically, the SL decoction significantly reduced elevated FPG levels (*p* < 0.05), and both the HL and SL decoctions improved insulin levels (HL: *p* = 0.278; SL: *p* = 0.053). These findings suggest that the antibacterial effects of SL decoction reduce gut microbiota diversity and richness, allowing beneficial bacteria more space to thrive, ultimately regulating the gut microbiota and exerting hypoglycemic effects.

The interaction between TCM formulations and gut flora is not an isolated phenomenon. Another study demonstrated that ginsenoside Rb1, a protopanaxdiol extracted from the root of *Panax quinquefolius* L. (Araliaceae; Panax), could alleviate diabetes by influencing the gut microbiota (Zhou et al. 2023). This effect occurs alongside other mechanisms such as glucose transporter type 4 (GLUT4) translocation (Shang et al. 2007), adenosine monophosphate (AMP) activation (Shen et al. 2015), and release of free fatty acids (Yu et al. 2015a). Furthermore, a type of tea polysaccharide extracted from Wuyi rock tea coarse tea powder has been found to address glucose metabolism disorders and their complications by regulating the intestinal flora, rather than influencing the activity of key enzymes in glucose metabolism, protecting pancreatic islet cells, promoting insulin secretion (Li et al. 2015), or through antioxidant activity (Chen et al. 2005; Fan et al. 2018).

The anti-diabetic effects of several TCMs have been observed, particularly in addressing insulin resistance. In a seven-week study, two groups received different concentrations of *Morus alba* fruit extracts: one with 50% ethanol (MFP50) and the other with 90% ethanol (MFP90), each at a dosage of 400 mg/kg. The results indicated significant reductions in FPG levels, with decreases of 31.9% in the MFP50 group and 47.5% in the MFP90 group, both compared to the diabetes model group (*p* < 0.01). Additionally, oral glucose tolerance test area under curve (OGTT-AUC) values were significantly lower in these groups than in the diabetes model group. Insulin levels (FINS) were significantly reduced in both the MFP50 (*p* < 0.05) and MFP90 (*p* < 0.01) groups relative to the diabetes model group. HOMA-IR values in the MFP90 group were also substantially lower than the metformin group, demonstrating that *Morus alba* fruit polysaccharides effectively improved insulin resistance by reducing FINS and HOMA-IR values in T2DM (Jiao et al. 2017).

In another four-week study, groups treated with different concentrations of Korean pine nut protein (PNP), PNP-low (L), PNP-medium (M), and PNP-high (H), exhibited significant improvements in HOMA-IR values compared to the diabetes model group (PNP-L: 6.96 ± 0.05; PNP-M: 5.05 ± 0.03; PNP-H: 6.20 ± 0.02 vs. DM: 8.94 ± 0.05) (*p* < 0.05). Similar effects have been observed with other TCMs, such as the aqueous extract of *Trichosanthes kirilowii* Maxim. (Cucurbitaceae; Trichosanthes) (TKE) (Lo et al. 2017) and combined extracts of *Scutellaria baicalensis* Georgi (Lamiaceae) (SR) and *Coptis chinensis* (CR) (Cui et al. 2019).

### Biological mechanisms involved in the protective effects of Chinese medicines against diabetes

**Figure 1.**
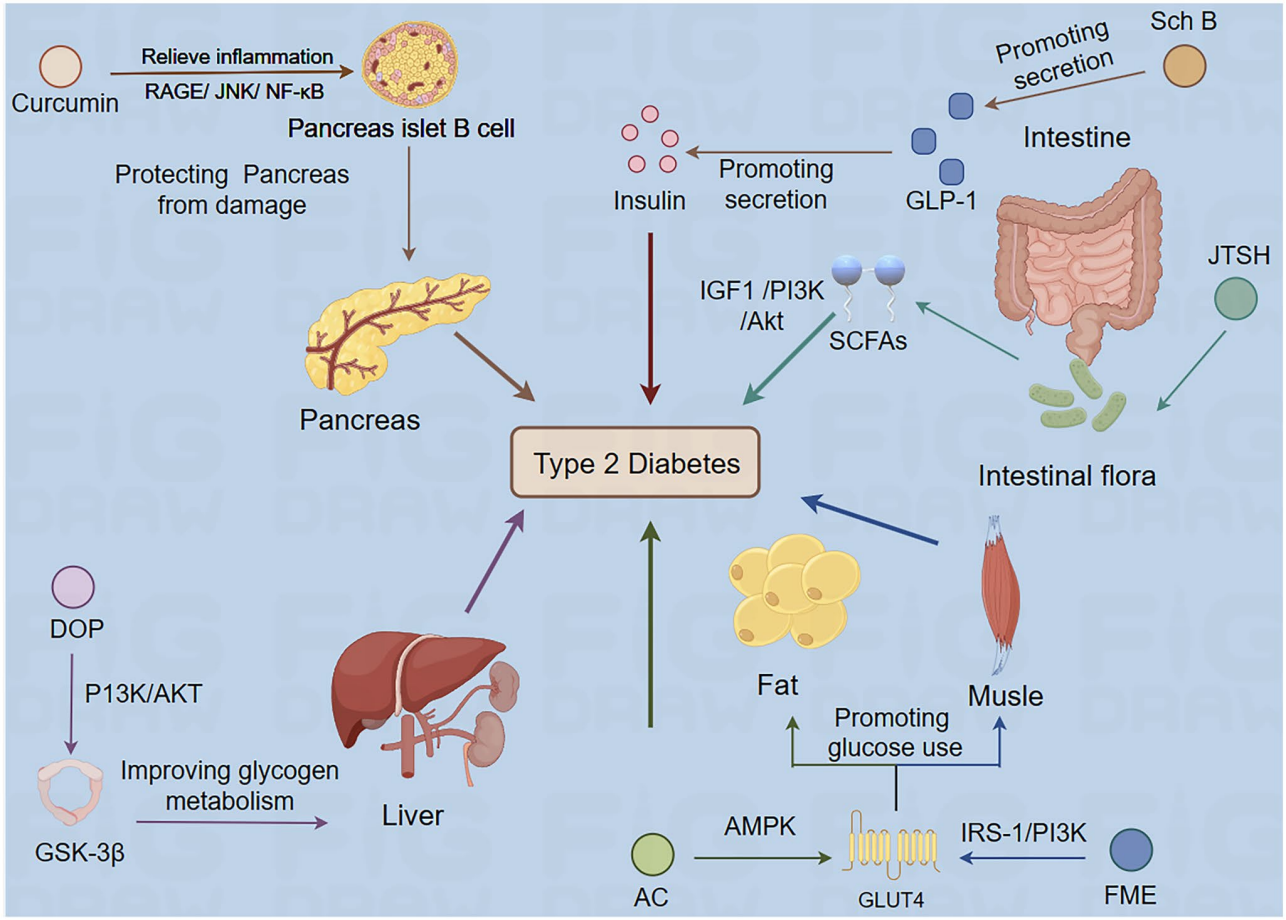
Biological mechanism of Traditional Chinese medicine treating type 2 diabetes mellitus (by Figdraw). Sch B: *Schisandrin* B; JTSH: Jiang Tang San Huang Wan pill; FME: *Folium Mori* extract; AC: *Antrodia Camphorata*; DOP: *Dendrobium officinale* polysaccharide.

The biological mechanisms underlying the efficacy of TCMs in treating diabetes are closely aligned with contemporary pharmacological theories on the pathogenesis and therapeutic targets of diabetes mellitus. This alignment is shown in Figure 1. The following sections summarize research studies on TCMs in the treatment of T2DM, with a particular focus on elucidating their biological mechanisms.

#### Promoting the transport and use of glucose

GLUT4 is essential in regulating glucose uptake by facilitating the translocation of glucose transporters from intracellular vesicles to the plasma membrane in response to insulin or non-insulin stimuli such as exercise and hypoxia. Disruption in GLUT4 function and content alters glucose homeostasis. Insulin binding to the insulin receptor (InsR) on target cells initiates a cascade of signaling events that activate various molecules, including the insulin receptor substrate (IRS), phosphoinositide 3-kinase (PI3K), and AKT. Activation of AKT leads to phosphorylation of its substrates, enhancing tissue glucose utilization by modulating GLUT4 activity (Stumvoll et al. 2005; Cheng et al. 2010). Additionally, the adenosine 5′-monophosphate (AMP)-activated protein kinase (AMPK) signaling pathway contributes to GLUT4 translocation to the plasma membrane, influencing glucose uptake (Li et al. 2021). Active components in some traditional Chinese and herbal medicines have been shown to have hypoglycemic effects by affecting these glucose metabolisms and transport-related signaling pathways.

Anemoside B4, a monomeric natural compound isolated from *Pulsatilla chinensis* (Bge.) Regel (Ranunculaceae) has been reported to exhibit hypoglycemic effects by modulating GLUT4 in diabetic rats (Gong et al. 2023). Studies indicate that B4 can enhance PI3K/AKT phosphorylation both *in vivo* and *in vitro*, indirectly boosting GLUT4 protein expression (Gong et al. 2023). SB-EtOAc significantly stimulates GLUT4 translocation in L6 cells and markedly improves glucose uptake by activating the AMPK-GLUT4 signaling pathways (Huang et al. 2016). In another study, Jiao-Tai-Wan (JTW) increased glucose consumption in adipocytes, partly by improving the expression and translocation of GLUT4 *via* the AMPK pathway (Yuan, Tang, et al. 2019).

*Scutellaria baicalensis* (SR) and *Coptis chinensis* (CR) have been used in combination for thousands of years in clinical practice to treat T2DM. They are known to significantly influence key indicators of the insulin signaling pathway, such as GLUT2, PI3K, and Akt, which play a crucial role in liver insulin-activated glucose uptake and inhibition of glucose release from hepatocytes. A study by Cui et al. (2018) found that low and high concentrations of SR and CR (LSC and HSC) significantly upregulated the expression of mRNA and proteins of PI3K, Akt2, and GLUT2.

After food ingestion, the liver absorbs approximately one-third of the ingested glucose. The remainder is absorbed primarily into skeletal muscle through insulin-dependent mechanisms. Skeletal muscle insulin resistance is characterized by reduced insulin-stimulated glucose uptake, leading to persistently high blood glucose levels and inadequate energy supply. Mulberry leaf, derived from *Morus alba* is a traditional Chinese medicine ingredient used to treat diabetes. Research has shown that FME, a flavonoid and polyphenolic component of mulberry leaf, exhibits significant anti-hyperglycemic activity by activating the skeletal muscle insulin receptor substrate (IRS)-1/PI3K/GLUT-4 signaling pathway (Cai et al. 2016). During a 4-week period, diabetic rats administered FME exhibited significant reductions in body weight and improvements in abnormal fat accumulation in skeletal muscle (Cai et al. 2016).

Recent research has underscored the effectiveness of AVI, a triterpenoid saponin extracted from *Dipsacus asper* Wall. ex Henry (Caprifoliaceae), in improving insulin resistance in skeletal muscle associated with T2DM. AVI has been found to activate the AMPK signaling pathway and the insulin-like growth factor 1 receptor (IGF1R) signaling pathway within skeletal muscle. It also upregulates mitochondrial function, balances energy metabolism, and improves glucose uptake disorders in insulin-resistant tissues and cells (Shu et al. 2024). Additionally, the Chinese Tang-Min-Ling (TML) formula has been clinically observed to positively regulate glucose metabolism in patients with T2DM (Tong et al. 2013). TML effectively regulates AMPK and GLUT4, thus enhancing the skeletal muscle response to insulin. This mechanism may be a crucial pathway for TML to improve insulin resistance and glucose metabolism (Zhen et al. 2011).

Insulin signaling plays a pivotal role in regulating glucose and insulin metabolism in adipose tissue, influenced by IRS-1 tyrosine phosphorylation and translocation of GLUT4. *Antrodia camphorata* (Taiwanofungus) (AC), a valuable medicinal fungus native to Taiwan’s forests, has been shown to significantly contribute to insulin sensitization in animal models of insulin resistance. Studies indicate that AC enhances the expression of IRS-1, PI3K, and GLUT4 in adipose tissue (Chung et al. 2018). Previous reports suggest that AC may enhance the insulin pathway by stimulating AMPK, which increases GLUT4 translocation, and by activating peroxisome proliferator-activated receptor alpha (PPARα), leading to a reduction in plasma free fatty acid levels. These actions collectively result in hypoglycemia and improved insulin resistance (Kuo et al. 2015a, 2015b).

A study of the flavonoid component (FC) of *Agrimonia pilo*sa Ledeb. (Rosaceae) revealed its efficacy in improving glucose metabolism in models of insulin resistance. FC enhanced insulin sensitivity in adipocytes and adipose tissue by alleviating disrupted oxidative homeostasis and reducing the expression and secretion of inflammatory cytokines, acting through the Jun N-terminal kinase (JNK)/PI3K/Akt pathway (Guo et al. 2023).

In the context of adipocytes, the G protein-coupled receptor 120 (GPR120) plays a crucial role in regulating adipogenesis, inflammation, glucose uptake, and insulin resistance, making it a promising target for treating metabolic disorders. GPR120 has been shown to increase glucose uptake in 3T3-L1 preadipocytes by activating the PI3K-AKT pathway and facilitating GLUT4 translocation (Oh et al. 2010). Vaccarin activates GPR120. This activation triggers the PI3K/AKT/GLUT4 signaling pathway in 3T3-L1 preadipocytes, leading to GLUT4 translocation and activation of the insulin signaling pathway, promoting glucose uptake in these cells (Jia and Liu 2022).

The forkhead box O1 (FoxO1) plays a critical role in glucose metabolism and gluconeogenesis by regulating the transcription of genes such as phosphoenolpyruvate carboxykinase (PEPCK), G6Pase, and glucokinase (GK) through promoter binding (Ganjam et al. 2009). Recent studies have indicated that specific Chinese herbal prescriptions can modulate FOXO1 activity. For example, the anti-diabetic effect of the Chinese herbal formula ZQR is associated with regulating the expression of PEPCK, G6Pase, and GK at the molecular level through the suppression of FOXO1 in the liver (Huang et al. 2012). Another study highlighted the hypoglycemic mechanism of resveratrol, which promotes the deacetylation of FoxO1. This deacetylation enhances the ability of FoxO1 to displace hepatic nuclear factor 4 (HNF-4) from its proximal promoter binding site, ultimately leading to suppression of GK gene expression (Ganjam et al. 2009).

Huanglian-Renshen-decoction (HRD), a classic traditional Chinese herbal medicine, has been used in clinical settings to treat diabetes for centuries and has been shown to be effective (Yuan, Wang, et al. 2019). A network pharmacology study suggested that the PI3K/Akt/FoxO1 signaling pathway might be involved in the ability of HRD to inhibit liver glucose production, and further experimental results have confirmed the validity of this mechanism (Wu et al. 2021).

In addition to modulating well-known insulin signaling pathways such as PI3K/AKT and AMPK, TCM also affects pathways related to extracellular regulated protein kinases (ERK) and hypoxia-inducible factor-1α (HIF-1α). The AKT pathway, also known as the protein kinase B (PKB) system, plays a crucial role in the development of various metabolic diseases. Additionally, protein kinase A (PKA), integral to the G-protein-coupled receptor signaling pathway, is vital in glucose metabolism. The PKA substrate, ERK, is closely associated with HIF-1α. Research indicates that the Si Wei Jiang Huang Tang San (SWJHTS) TCM formula can promote glucose consumption by activating the ERK signaling pathway and inhibiting HIF-1α, contributing to improved diabetes management (Xu et al. 2023).

#### Improving glycogen metabolism

The liver plays a crucial role in maintaining normal glucose levels, producing glucose during fasting periods and storing it postprandially. Insulin regulates these processes by inhibiting glycogen breakdown and stimulating glycogen synthesis, reducing glucose production, and enhancing glucose metabolism. However, in T2DM, hepatic processes become dysregulated due to insulin resistance in the liver. This resistance leads to higher rates of glycogenolysis and gluconeogenesis, causing the liver to release excess glucose into the bloodstream, contributing to hyperglycemia (Huang et al. 2018).

Insulin primarily regulates blood glucose levels by signaling through the PI3K/Akt pathway. Upon food consumption, insulin produced by pancreatic beta cells enters the bloodstream and activates the insulin receptor on liver cell membranes. This activation initiates the phosphorylation of the tyrosine site on the insulin receptor substrate-1/2 (IRS-1/2), which is crucial for insulin’s metabolic effects in the liver. Subsequently, PI3K is activated, leading to downstream biological metabolism regulation by Akt. Several traditional Chinese herbs have been shown to influence hepatic glycogen synthesis and degradation pathways, demonstrating hypoglycemic effects.

*Dendrobium officinale* polysaccharide (DOP), a biomacromolecule isolated from the roots of *Dendrobium officinale* Kimura et Migo (Orchidaceae), has shown significant therapeutic benefits in BALB/c mice with T2DM, induced by streptozotocin (STZ). Treatment with DOP led to notable reductions in FPG, OGTT results, and HOMA-IR levels. It increased the hepatic glycogen content. These effects were partially achieved by regulating the expression of glycogen synthase kinase 3 beta (GSK-3β), glycogen synthase, and GLUT4 in the liver or muscle, primarily through the PI3K/Akt signaling pathway (Wang et al. 2018).

Another study demonstrated that DOP significantly inhibited the glucagon-mediated cAMP-PKA pathway. This inhibition resulted in an increase in glycogen synthase expression, a decrease in glycogen phosphorylase expression, and promoted hepatic glycogen synthesis while inhibiting hepatic glycogen degradation in diabetic mice. DOP was found to produce more stable α-particles within the structure of diabetic glycogen. This structural stability reduces binding to glycogen phosphorylase, slowing some degradation processes (Liu et al. 2020).

Similarly, a polysaccharide extracted from another *Dendrobium* species, *Dendrobium huoshanense* C.Z.Tang et S.J.Cheng (DXG), has also been shown to enhance hepatic glycogen storage and reduce hepatic glucose levels in T2DM mice. Like the positive control drug metformin, DXG was found to inhibit hepatic glucose production by increasing the phosphorylation of IRS-1, PI3K, and Akt (Wang, Li, et al. 2019).

Treatment with RPE, a polyphenol-enriched extract of *Rosa rugosa* Thunb. (Rosaceae), has demonstrated significant therapeutic effects in diabetic rats. It resulted in a substantial increase in phosphorylated insulin receptor substrate-1 (p-IRS-1), insulin receptor (p-IR), protein kinase B (p-AKT), and glycogen synthase kinase-3 beta (p-GSK-3β). These increases lead to marked improvements in glycogen synthesis and blood glucose regulation (Liu et al. 2017). RPE has been found to inhibit the activity of α-glucosidase, an enzyme that breaks down complex carbohydrates into absorbable monosaccharides in the small intestine. By inhibiting this enzyme, RPE can decrease carbohydrate absorption, effectively reducing postprandial blood glucose levels and thus aiding in the management of blood glucose.

BCLE, an ethanol extract derived from *Belamcanda chinensis* (L.) DC. (Iridaceae; Belamcanda), has exhibited hypoglycemic effects in C57BL/6 J mice T2DM. BCLE reduces hepatic levels of GSK-3β, enhancing glycogen synthesis and glucose transport, which ameliorates hyperglycemia (Guo et al. 2019). In a separate study (Woźniak and Matkowski 2015), a specific pectic polysaccharide (FPLP) was extracted and purified from the fruits of *Ficus pumila* L. (Moraceae). This study demonstrated that the hypoglycemic mechanism of FPLP is related to improved liver glycogen metabolism, which is associated with the activation of the insulin signaling pathway along the IRS-1/PI3K/Akt/GSK-3β/GS axis and the regulation of major enzymes involved in liver glycogen metabolism, including glucokinase, PEPCK, and G6Pase (Wu et al. 2017).

The Sanggua drink (SGD) has been shown to enhance the expression of IRS-2, PI3K, and AKT gene**s** and protein**s** in the liver of diabetic rats. This leads to increased hepatic glycogen production and a hypoglycemic effect (Cai et al. 2018). Another study demonstrated that TPX, derived from the mangiferin derivative 1,3,6,7-tetrapropylene acyloxy ketone, targets AMPK and PI3K/AKT. This targeting restores the insulin signaling pathway, increases liver glycogen synthesis, and potentially protects insulin-resistant hepatocytes from glucose metabolism disorders (Fan et al. 2023).

*Salvia divinorum* Epling & Jativa (Lamiaceae), a native herb of Mexico, contains tanshinone as its main pharmacologically active ingredient. A study investigating the effects of tanshinone I on T2DM and its potential mechanisms found that tanshinone I treatment led to a decrease in the phosphorylation of total cholesterol, triglycerides, LDL-C, non-esterified fatty acids (NEFA), and IRS-1 at Ser307. It resulted in an increase in body weight, a reduction in blood glucose levels, and an alleviation of insulin resistance in T2DM rats (Wei et al. 2017).

In recent studies, a purified RG polysaccharide (RGP) extracted from *Rehmannia glutinosa* Libosch. (Orobanchaceae) has been identified as a potential therapeutic option for type 1 diabetes. These studies highlighted that the anti-diabetic properties of RGP are correlated with increased pancreatic insulin synthesis and secretion, increased liver glycogen synthesis, and reduced hepatic gluconeogenesis. RGP, similar to metformin, can rectify gluconeogenesis disorders in the liver, thus improving glucose metabolism in diabetic mice (Zhou et al. 2015).

#### Promoting GLP-1 release

GLP-1, secreted from the distal ileum and cells located in the rectum and colon, is particularly favored by researchers for its glucose-dependent actions, the absence of hypoglycemic response, and significant cytoprotective functions. When GLP-1 binds to its receptors, it increases intracellular cAMP levels and activates protein kinase A. This activation enhances the transcription and translation of the insulin gene in β cells, increasing their sensitivity to glucose stimulation signals and consequently increasing insulin secretion. Modern pharmacological studies have shown that some Chinese herbs can treat diabetes by promoting GLP-1 secretion, thus effectively regulating blood glucose levels.

ACE, the ethyl acetate fraction of *Acorus calamus* L. (Acoraceae), has shown antidiabetic activity. It is believed to activate the Wnt signaling pathway, improve the gene expression of proglucagon and prohormone convertase 3, leading to incretin and islet protection, and decrease blood glucose levels by either directly or indirectly increasing GLP-1 secretion (Liu et al. 2015). Additionally, cell membrane chromatography (CMC), a novel affinity chromatography technique for studying receptor-drug interactions, has been utilized in recent research. A study using this technique discovered that schisandrin B (Sch B), a small molecule, exhibits a high affinity for GLP-1 receptor, suggesting that Sch B could potentially act as a GLP-1 receptor agonist.

*Arctium lappa,* known as ‘Niubangzi’ in China, is recognized in the Chinese pharmacopeia for its traditional therapeutic uses. A study indicated that total lignans from the ethanol extract of *Arctium lappa* (TLFA) demonstrate a significant hypoglycemic potential in GK rats. Hypoglycemic effects involve stimulation of insulin secretion, promotion of GLP-1 release, and reduction of intestinal glucose absorption (Xu et al. 2014). Similarly, L-SGgly, an extract of Luohanguo (LHG), can potentially improve hyperglycemia and regulate insulin secretion by increasing GLP-1 levels in T2DM rats (Zhang et al. 2020).

Berberine, the primary active component of *Coptis chinensis* Franch, has been used effectively in the treatment of diabetes mellitus. A proposed mechanism for the hypoglycemic effect of berberine involves the induction of GLP-1 secretion (Lu et al. 2009; Yu et al. 2010). Recent research has further elucidated that berberine-mediated GLP-1 secretion plays a significant role in regulating glucose homeostasis. This study also suggested that TAS2R38, a bitter taste receptor in the gastrointestinal tract, is involved in the GLP-1 secretion process induced by berberine, highlighting a novel interaction between dietary components and metabolic regulation (Yu et al. 2015b).

#### Protecting pancreatic islets from damage

The primary function of pancreatic beta cells is to secrete insulin, which plays a crucial role in regulating blood glucose levels. In T2DM, elevated blood glucose levels can lead to beta cell dysfunction, characterized by inadequate insulin secretion and the resultant hyperglycemia. Over time, this dysfunction often triggers a compensatory increase in insulin production, which further strains the β-cells, leading to their gradual deterioration. This decline in beta cell function not only impairs glucose regulation but also exacerbates the progression of T2DM. Ultimately, beta cells may not produce sufficient insulin, resulting in an absolute deficiency. Several factors contribute to β-cell apoptosis, including hyperglycemia, hyperlipidemia, activation of pro-inflammatory factors, and oxidative stress (Choi and Woo 2010). Therefore, protecting pancreatic β-cells and inhibiting their apoptosis are vital strategies in managing T2DM.

Research on the treatment of diabetes mellitus with Chinese herbs has indicated that certain herbs can exert a hypoglycemic effect by enhancing the function of pancreatic islets. These herbs act by counteracting the factors that lead to β-cell apoptosis and dysfunction. They play a significant role in improving blood glucose regulation and overall diabetes management.

Several studies have established a correlation between β-cell dysfunction and oxidative stress, which is often induced by hyperglycemia and hyperlipidemia (Drews et al. 2010). In patients with T2DM, persistently high blood sugar levels lead to glucose oxidation and protein glycosylation, resulting in ROS overproduction (Koeck et al. 2009). *Citrullus lanatus* (Thunb.) Matsumu.et Nakai (Cucurbitaceae; Citrullus), a watermelon species cultivated in China, has shown promising results in diabetes management. An animal study reported that Sanbai melon seed oil (SMSO) significantly reduced blood glucose levels, increased plasma insulin, repaired islet tissue damage, and increased antioxidant activity in diabetic rats, demonstrating its potent anti-diabetic effects. The mechanism underlying SMSO efficacy may involve Nrf3 induction through the Akt/GSK-3β-mediated pathway, which in turn stimulates the expression of antioxidant proteins and prevents high glucose-induced oxidative stress in T2DM rats (Wang, Xi, et al. 2019).

*Anemarrhena asphodeloides* Bge. (Liliaceae; Anemarrhena) extract (AAE) was shown in another study to restore pancreatic islet cell function by upregulating PRDX4 expression (Yan et al. 2021). PRDX4 is an antioxidant enzyme crucial for organ protection during oxidative stress, and its overexpression may protect pancreatic β-cells from streptozotocin-induced injury (Yamada et al. 2012). Various Chinese remedies, including the Shenqi compound (SQC), Acorn (*Quercus liaotungensis* Koidz (Fagaceae; Quercus)), and Jiu Huang Lian (*Coptis chinensis* steamed with rice wine, JHL), have been shown to contribute significantly to T2DM treatment. These remedies alleviate oxidative stress in pancreatic tissue, specifically targeting and protecting β-cell function (Li et al. 2013; Xu et al. 2021; Yang et al. 2023).

Inflammation is a key pathological factor that contributes to insulin resistance, disrupts glucose metabolism, and accelerates the progression of diabetes. Recent studies have highlighted the significant role of advanced glycation end-products (AGEs) and their receptor (RAGE) in the development of diabetes mellitus, particularly in the damage to pancreatic β-cells (Kay et al. 2016). Increased expression and activation of RAGE are responsible for triggering inflammation, toxicity, and apoptosis in pancreatic β-cells (Adami et al. 2004). Activation of RAGE-related pathways may lead to overexpression of inflammatory factors and peroxides through the JNK/NF-κB signaling pathway, which is involved in organ damage.

Curcumin, an acidic polyphenolic compound derived from the Chinese herb turmeric, has been shown to play a significant role in improving glucose metabolism and protecting pancreatic β-cells in a rat model of T2DM. This protection is achieved through its anti-inflammatory and anti-oxidative stress effects (El-Azab et al. 2011). Further research has elucidated the potential mechanism behind the anti-inflammatory and anti-apoptotic effects of curcumin in pancreatic β-cells. A study revealed that curcumin inhibits the RAGE/JNK/NF-κB pathway, thus mitigating the harmful effects associated with inflammation and apoptosis in these cells (Qihui et al. 2020).

Under normal physiological conditions, the populations of pancreatic β-cells are maintained through a dynamic balance regulated by islet apoptosis, proliferation, and ductal insulin production. However, excessive rates of β-cell apoptosis can precipitate diabetes (Pazdro and Burgess 2010). There are two main pathways of apoptosis: the intrinsic pathway, which is driven by mitochondria, and the exogenous pathway, which is mediated by receptors (Emamaullee and Shapiro 2006). Research has shown that PAGR, a purified anthraquinone-glycoside from *Rheum palmatum* L. (Polygonaceae), has hypoglycemic properties and can attenuate T2DM. The mechanism behind the effectiveness of PAGR includes reducing oxidative stress by improving lipid metabolism and increasing antioxidant capacity. This reduction in stress mitigates damage to mitochondrial structures and down-regulates the activation of the mitochondrial-induced cell death pathway, inhibiting β-cell apoptosis and enhancing β-cell function. Additionally, PAGR minimizes β-cell apoptosis by reducing Fas/FasL-mediated apoptosis in pancreatic tissue (Cheng et al. 2019).

A study on the role of quercetin in treating T2DM revealed that quercetin prevents palmitate-induced apoptosis. It achieves this by inhibiting caspase-3, −9, and −12 activation, increasing the B-cell lymphoma-2 (Bcl)-2/(BCL-2-associated X) BAX ratio, and restoring damaged mitochondrial membrane potential (Zhuang et al. 2018).

In addition to protecting β-cells, Chinese herbs have also been found to exert beneficial effects on α-cells. Cyclin-dependent kinase 4 (CDK4) plays a crucial role in mammalian cell proliferation and is considered a key target in α-cell proliferation. Clinical studies have demonstrated that Gegen Qilian decoction (GQD) has significant hypoglycemic effects (Tong et al. 2011). GQD promotes α-cell proliferation by restoring the expression of the Cdk4 and Irs1 genes. Recent research has highlighted the potential of α-cells as a viable source of new β-cells, which could replace lost or dysfunctional β-cells in patients with type 1 and type 2 diabetes. This advancement provides a promising avenue for diabetes gene therapy, utilizing reprogramming strategies to generate β-cells from α-cells, thereby offering new therapeutic potentials for diabetes management (Xu et al. 2022).

##### Improving intestinal flora

Abnormal intestinal flora function and composition are key environmental determinants in the development of insulin resistance and T2DM (Chen et al. 2019). The disturbed gut flora produces peptidoglycans and lipopolysaccharides, which released into the bloodstream from the intestinal lumen, contribute to insulin resistance and the onset of T2DM (Yang et al. 2017). There is significant evidence that the abundance and diversity of the intestinal flora is altered in patients with T2DM (Zhang et al. 2013), highlighting the importance of targeting improvement of the gut flora as a strategic therapeutic approach to manage this disease. Research in Chinese medicine has identified many herbs that can exert a hypoglycemic effect by improving intestinal flora, including studies published throughout various years (Gao et al. 2018; Gong et al. 2021; Wang et al. 2021; Xia et al. 2021; Sun et al. 2021a; Zhang et al. 2022; Chen et al. 2023; Tawulie et al. 2023).

*Polygonatum sibiricum* (Liliaceae; Polygonatum) polysaccharide (PP), a primary component of *Polygonatum sibiricum*, has been found to positively influence gut flora. PP interventions have increased beneficial genera, such as *Turicibacter*, and decreased harmful genera, including two genera of the family Lachnospiraceae and the genus *Romboutsia* (Chen et al. 2023). The Qijian mixture, a new combined TCM prescription to treat T2DM, has been shown to induce structural changes in intestinal flora. These changes are correlated with the antidiabetic effects of the Qijian mixture, particularly by enriching beneficial bacteria such as the firmicutes and bacteroidetes in the gut (Gao et al. 2018).

*Astragalus* is one of the most widely used herbs in TCM to treat T2DM, with Astragaloside IV (AS-IV) being one of its important bioactive components. A study confirmed the hypoglycemic effect of AS-IV *in vitro* and *in vivo*, revealing its ability to balance the abundance of gut flora. In addition to scavenging ROS and balancing the structure of intestinal flora, AS-IV is metabolized into cycloastragenol (CAG) by intestinal bacteria. This metabolism further reduces SGLT2 expression and inhibits glucose transport, thus contributing to improvements in T2DM (Gong et al. 2021). Furthermore, *Dendrobium officinale* has also been shown to significantly reduce FPG levels and regulate intestinal flora. This regulation particularly affects key bacterial taxa such as *Akkermansia* and *Parabacteroides*, which are associated with the development of T2DM (Wang et al. 2021).

Jiang Tang San Huang pill (JTSH) is a clinically used TCM formulation in the treatment of T2DM. JTSH has been found to modulate intestinal dysbiosis by preferentially increasing the activity of bile salt hydrolase (BSH) of certain bacteria (*Bacteroides*, *Lactobacillus*, and *Bifidobacterium*). This increase leads to the accumulation of unbound ileal bile acids, further upregulating the intestinal farnesoid X receptor/fibroblast growth factor 15 (FXR/FGF15) and TGR5/GLP-1 signaling pathways (Tawulie et al. 2023). Similarly, Coix seed polysaccharides have been shown to increase the level of short-chain fatty acids (SCFAs) through the intestinal flora. This increase activates the IGF1/PI3K/Akt signaling pathway, producing a hypoglycemic effect (Xia et al. 2021).

Insulin resistance can also be caused by a breakdown in the gut barrier, which allows lipopolysaccharide (LPS), a metabolite of gut bacteria, to enter the bloodstream. Studies have shown that vaccarin may protect the intestinal barrier by inhibiting the ERK/myosin light chain kinase (ERK/MLCK) signaling pathway. This inhibition helps counteract inflammation caused by intestinal leakage and subsequent release of endotoxins into the bloodstream, potentially reducing insulin resistance (Sun et al. 2021a). Similarly, the Shenqi compound intervention has been found to improve the structure and function of the rat intestinal barrier. This improvement is achieved by promoting the expression of Zonula occludens-1 (ZO-1), a critical protein for maintaining tight junction integrity, and by reducing LPS levels, further stabilizing the gut barrier and mitigating inflammatory responses (Zhang et al. 2022).

## Discussion

The main drugs used to treat diabetes include biguanides, sulfonylureas, glinides, glucosidase inhibitors, thiazolidinediones, and insulin analogues. However, the efficacy of these drugs does not meet all clinical needs. From the perspective of efficacy, some drugs exhibit poor or unsustainable effectiveness, significant fluctuations in blood glucose control, low compliance rates, and inadequate management of complications (Cannon et al. 2018). From the point of view of adverse reactions, these drugs, based on their different mechanisms of action, can cause unique side effects. For example, sulfonylureas and insulin medications may induce hypoglycemia (Unger 2012), GLP-1 receptor agonists can cause gastrointestinal reactions (Shetty et al. 2022), and SGLT-2 inhibitors are associated with urinary or reproductive system infections (Shi et al. 2022).

Given the safety and broad market prospects of TCM, researchers are increasingly focused on developing TCM as a treatment for T2DM. Chinese herbal medicine has demonstrated significant protective effects against diabetes. The potential of these drugs for diabetes treatment has been explored in various clinical trials at different stages. Studies have indicated that TCM can lower blood glucose levels, decelerate the transition from pre-diabetes to diabetes, reduce the risk of diabetic complications by controlling risk factors, and slow the progression of these complications. Mechanistic studies have shown that TCM can achieve hypoglycemic effects through multiple pathways, including promoting glucose transport and utilization, enhancing glycogen synthesis in the liver, inhibiting glycogenolysis, stimulating GLP-1 secretion, protecting pancreatic islet cells, and regulating intestinal flora.

However, the broader adoption of TCM as a primary therapy for treating and managing diabetes requires more well-designed and rigorously conducted clinical trials. These trials should adhere to the general principles of quality assurance, including multicenter collaboration, adequate sample sizes, randomized grouping, blinding, adherence to Good Clinical Practice (GCP), and compliance with human research ethics. Moreover, considering the multi-component and multi-target nature of Chinese medicines, the use of purified single bioactive compounds or mixed bioactive ingredients may offer more reliable insight into their clinical efficacy. Quality control is critical; the composition and stability of herbal medicines must be maintained consistently throughout the trials. The appropriate dosages and duration of treatment should be established based on preclinical studies.

The safety of Chinese medicines is paramount. Rigorous safety monitoring both before and during clinical trials is essential to ensure that these treatments are not only clinically effective but also well-tolerated and safe. This approach will help to establish a more robust scientific basis for the use of TCM in diabetes management, which could lead to greater acceptance and integration into mainstream healthcare systems.

## Conclusions

TCM should be used to explore the prevention and treatment of T2DM. This exploration should include investigating new theories of T2DM pathogenesis, identifying potential targets for screening drug candidates, and generating innovative ideas for the development of novel T2DM treatments. Beyond studying the therapeutic effects of TCM, it is equally crucial to consider the ‘druggability’ of these treatments for T2DM. This consideration should focus on developing new dosage forms of TCM and addressing the challenge of low bioavailability observed in some TCM compounds. By addressing these challenges, we can maximize the therapeutic potential of TCM in the management and treatment of T2DM, potentially leading to more effective and accessible treatment options for patients.

## Author’s contributions

The review was designed and performed by Xuansheng Ding, Yadong Ni, Xianglong Wu, Wenhui Yao, Yuna Zhang, and Jie Chen. The manuscript was written by Yadong Ni and Xianglong Wu and reviewed by Xuansheng Ding and Wenhui Yao. All authors have read and approved the final manuscript.
